# Conditional embryonic lethality to improve the sterile insect technique in *Ceratitis capitata *(Diptera: Tephritidae)

**DOI:** 10.1186/1741-7007-7-4

**Published:** 2009-01-27

**Authors:** Marc F Schetelig, Carlos Caceres, Antigone Zacharopoulou, Gerald Franz, Ernst A Wimmer

**Affiliations:** 1 Department of Developmental Biology, Göttingen Center for Molecular Biosciences, Johann-Friedrich-Blumenbach-Institute of Zoology and Anthropology, Georg-August-University Göttingen, GZMB, Ernst-Caspari-Haus, Justus-von-Liebig-Weg 11, 37077 Göttingen, Germany; 2 USDA-APHIS OFFICE, 4Av. 12-62 Zona 10, 01010-Guatemala City, Guatemala; 3 Department of Biology, University of Patras, Patras, Greece; 4 International Atomic Energy Agency, FAO/IAEA Agriculture and Biotechnology Laboratory Entomology Unit, A-2444 Seibersdorf, Austria; 5 USDA/ARS, Center for Medical, Agricultural and Veterinary Entomology, Gainesville, FL 32608, USA

## Abstract

**Background:**

The sterile insect technique (SIT) is an environment-friendly method used in area-wide pest management of the Mediterranean fruit fly *Ceratitis capitata *(Wiedemann; Diptera: Tephritidae). Ionizing radiation used to generate reproductive sterility in the mass-reared populations before release leads to reduction of competitiveness.

**Results:**

Here, we present a first alternative reproductive sterility system for medfly based on transgenic embryonic lethality. This system is dependent on newly isolated medfly promoter/enhancer elements of cellularization-specifically-expressed genes. These elements act differently in expression strength and their ability to drive lethal effector gene activation. Moreover, position effects strongly influence the efficiency of the system. Out of 60 combinations of driver and effector construct integrations, several lines resulted in larval and pupal lethality with one line showing complete embryonic lethality. This line was highly competitive to wildtype medfly in laboratory and field cage tests.

**Conclusion:**

The high competitiveness of the transgenic lines and the achieved 100% embryonic lethality causing reproductive sterility without the need of irradiation can improve the efficacy of operational medfly SIT programs.

## Background

The Mediterranean fruit fly (medfly), *Ceratitis capitata *(Wiedemann; Diptera: Tephritidae), is one of the most devastating and economically important insect pests [1]. An effective biological and environment-friendly control of this pest is the sterile insect technique (SIT) [2]. The SIT reduces a pest population by mass release of reproductively sterile male insects into a wildtype (WT) population of the same species. This leads to the decrease of progeny by competition of sterilized males with WT males for WT females [3]. Thus, the sterilization of the pest species in SIT programs is of major importance and is commonly induced by radiation. However, the sterility and competitiveness are indirectly correlated [4]. In some programs therefore lower doses of radiation are used to generate lines which are more competitive even though only partially sterile. In preventional release programs, completely sterile flies are released into pest-free areas to avoid the establishment of invasive fruit flies and to control the constant problem of re-infestation [5]. These programs have to use 100% sterile flies to avoid a novel introduction of insect pests. However, the competitiveness of such flies is reduced due to the high dose of radiation required for complete lethality, which results in the expensive need of high numbers of males per field-release and a high frequency of such releases.

A first approach to cause reproductive sterility by transgene-based embryonic lethality without the need of radiation has been successfully shown in the non-pest insect *Drosophila melanogaster *[6]. The system is based on the transmission of a transgene combination that causes embryo-specific lethality in the progeny. To limit the effect of the transgenes to the embryonic stage, promoter/enhancers (P/Es) from cellularization-specifically-expressed genes drive the expression of the tetracycline-controlled transactivator (*tTA*). The expressed heterologous transactivator then activates the expression of the lethal effector gene *hid*^*Ala*5 ^[7] and leads to complete embryonic lethality in *D. melanogaster*. To generate suppressible, dominant lethality in medfly and at the same time restrict the effects of lethality to embryos, a direct transfer of the sterility system from *D. melanogaster *[6] to medfly was previously tried. The genomic integration of the driver construct carrying the *sry α *P/E from *D. melanogaster *into medfly was successful, but none of the transgene insertions expressed the system activator *tTA *at a detectible level [8]. We therefore concluded that the cellularization-specific P/E from *D. melanogaster *is not functional in medfly and that endogenous P/Es have to be used to generate such a system.

Here, we report the development of the first transgenic embryonic lethality system for medfly using an early embryonic lethal transgene combination. When transgenic males carrying this system are mated to WT females, all progeny die during embryogenesis without the need of radiation. Due to the complete lethality in embryonic stages no fruit damage from developing larvae would occur from progeny of WT females mated to transgenic males and no transgenes would ingress into the wild population. Moreover, males carrying this system are highly competitive in laboratory and field cage tests. After successful evaluation, a combination of this new embryonic lethal medfly system with a sexing system will become a powerful tool to improve SIT programs.

## Results

### Isolation of cellularization-specifically-expressed genes and their P/Es from medfly

**Figure 1:**
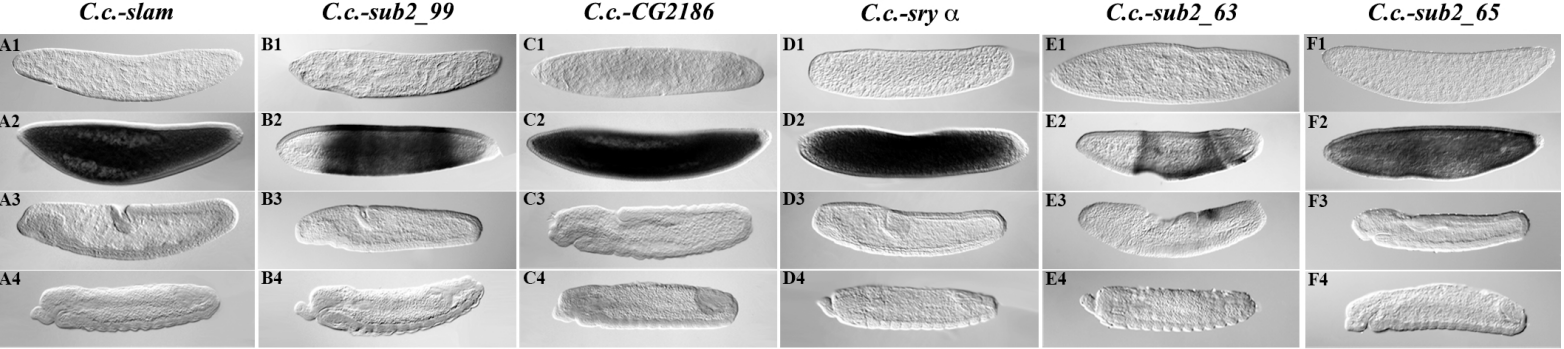
**Medfly genes expressed specifically during cellularization**. Gene expression is shown by WMISH on embryos from a 0–48 h egg collection of wildtype (WT) medflies with gene-specific RNA probes for different stages during embryogenesis: early blastoderm (×1), cellularization (×2), germ band elongation (×3) and germ band retraction (×4). The genes *C.c.-slam *(Ay), *C.c.-sub2_99 *(By), *C.c.-CG2186 *(Cy), *C.c.-sry α *(Dy), *C.c.-sub2_63 *(Ey), and *C.c.-sub2_65 *(Fy) are strongly expressed during cellularization (×2). *C.c.-sub2_63 *also showed expression during germ band elongation (E3). Gene names used in Schetelig et al. (2007) [9] correspond as follows: *sub1_68 = sub1_24 = C.c.-slam; sub1_478 = C.c.-CG2186*.

The isolation of the medfly homologs of the cellularization genes *sry α *and *nullo *by degenerate primer PCR using an embryonic cDNA pool was not successful [9]. Thus, we carried out PCR-based cDNA subtractions of different embryonic stages and identified several cellularization-specifically-expressed genes (Figure 1). The genes *C.c.-slow as molasses *(*C.c.-slam; *Figure 1A), *C.c.-sub2_99 *(Figure 1B), *C.c.-CG2186 *(Figure 1C), *C.c.-serendipity α *(*C.c.-sry α*; Figure 1D), *C.c.-sub2_63 *(Figure 1E), and *C.c.-sub2_65 *(Figure 1F) are expressed specifically during medfly blastoderm cellularization (Figure 1). None of the genes shows maternal expression or expression at later stages, except *C.c.-sub2_63*, which is additionally expressed during germ band elongation (Figure 1E3).

### Germline transformation with driver and effector constructs

By inverse PCR, we isolated the P/Es from *C.c.-slam, C.c.-sub2_99, C.c.-CG2186, C.c.-sry α*, and *C.c.-sub2_63 *containing about 0.4 to 1.9 kb of the complete 5'UTR and upstream sequences. The isolated P/Es were fused to the tetracycline-controlled transactivator gene *tTA *and used to engineer different driver constructs (*sl1-tTA*, *sl2-tTA*, *99-tTA*, *CG2186-tTA*, *sryα 2-tTA*, and *63-tTA*) embedded into *piggyBac *vectors carrying polyubiquitin (PUb)-driven DsRed as a germline transformation marker [10]. Additionally, three effector constructs were generated (*TREp-hid*^*Ala*5^, *TREhs43-hid*^*Ala*5^, and >*TREp-hid*^*Ala*5^>) carrying the lethal factor *hid*^*Ala*5 ^under control of either *P *element [11] or *hsp70 *basal promoters [12] from *D. melanogaster*. In the >*TREp-hid*^*Ala*5^> construct the lethality inducing transgene is flanked by *gypsy *insulator elements (> = *gypsy *element in 5'-3' orientation), which should reduce position effect-caused variable expression [13]. Except for *sl1-tTA*, all constructs carry a minimal *attachment P *(*attP*) site [14], which potentially enables site-specific *phiC31*-integrase-mediated integration to modify transgenes at successfully evaluated genomic positions [15].

**Figure 2:**
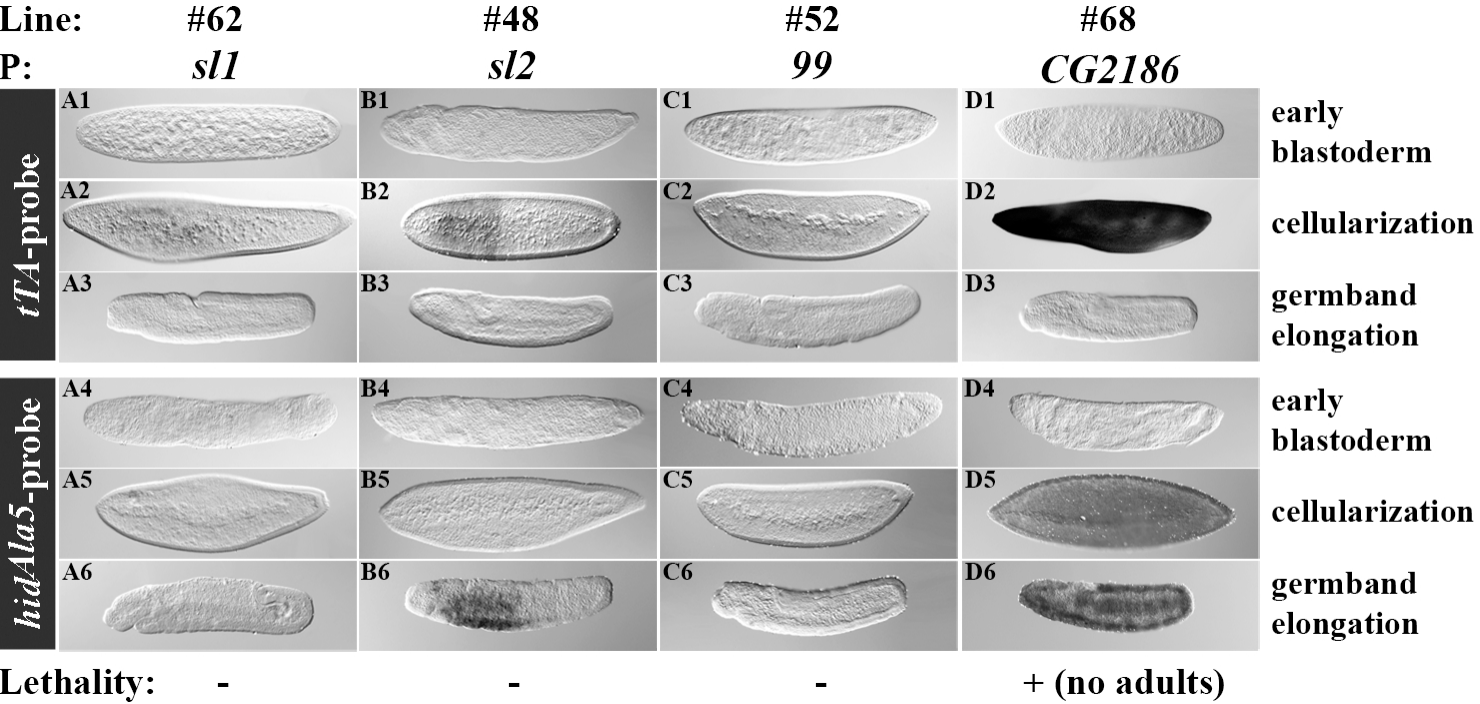
***tTA *and *hid*^*Ala*5 ^expression depends on different P/Es**. Expression of *tTA *and *hid*^*Ala*5 ^is shown by WMISH performed on embryos from a 0–48 h egg collection of medfly lines carrying both driver and effector constructs in homozygous condition. The developmental stages of embryogenesis are indicated to the right of the respective panels. The lines carry driver constructs with different P/E (P) driving the *tTA*. The depicted lines are representative for independent lines (three for *sl1*, two for *sl2*, three for *99*, and one for *CG2186*) carrying the respective driver construct. All presented lines derive from the effector line *TREhs43-hid*^*Ala*5^_F1m2 and were reared on Tc-free adult food. 100% lethality in lab tests is indicated with + and the stage of complete lethality is indicated in brackets.

**Figure 3:**
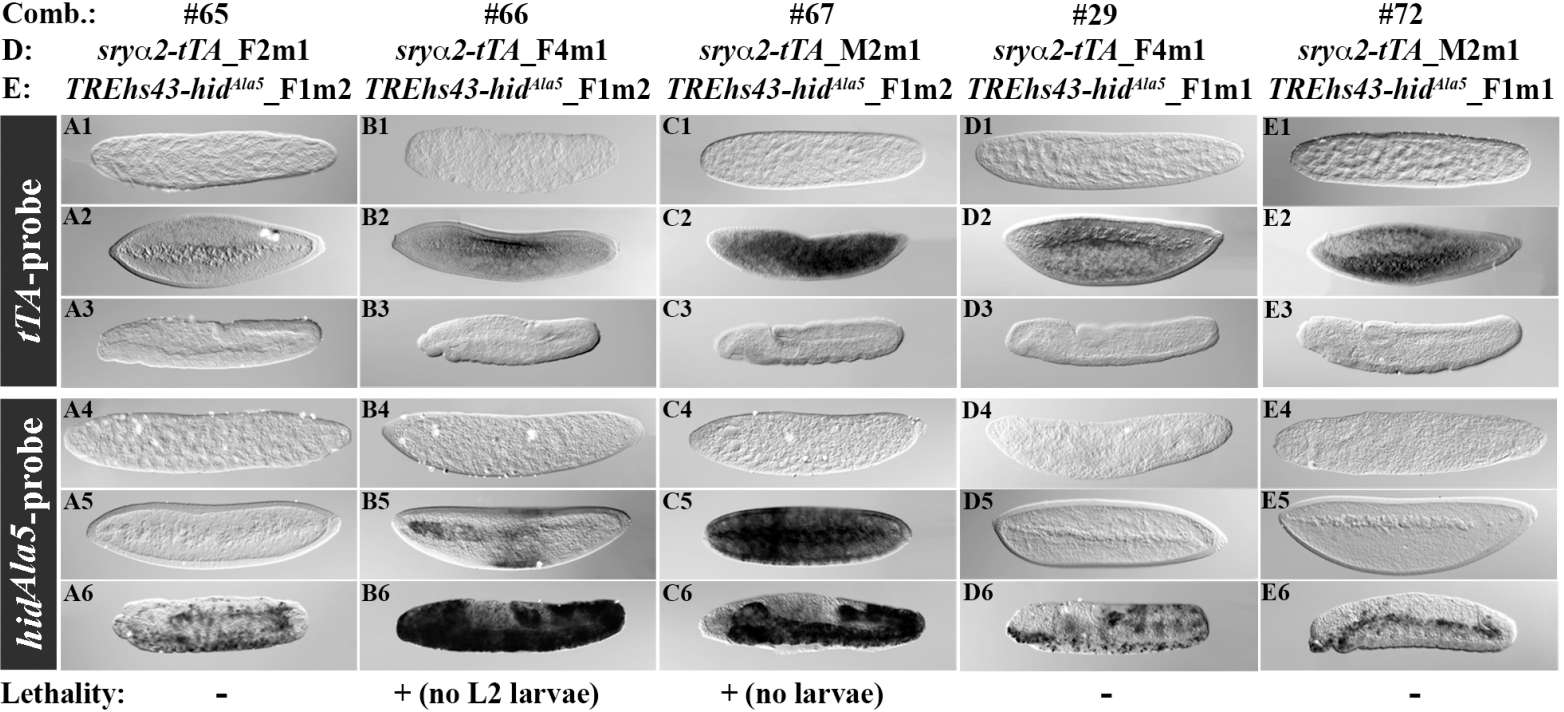
***tTA *and *hid*^*Ala*5 ^expression depending on different integration sites**. The expression of *tTA *and *hid*^*Ala*5 ^is shown by WMISH performed on embryos from a 0–48 h egg collection of medfly combinations (comb.) carrying both driver (D) *sryα 2-tTA *and effector (E) *TREhs43-hid*^*Ala*5 ^in heterozygous conditions. The developmental stages of embryogenesis are as indicated in Figure 2. The flies were reared on Tc-free adult food. 100% lethality in lab tests is indicated with + and the stage of complete lethality is indicated in brackets.

Five driver constructs (*sl1-tTA*, *sl2-tTA*, *99-tTA*, *CG2186-tTA*, and *sryα 2-tTA*) and the three effector constructs were used for germline transformation of medfly. For each construct we obtained transgenes of which we further analyzed a maximum of three independent lines (Figure 2 and Figure 3).

### Early expression of *tTA *incites *hid*^*Ala5*^-mediated lethality

We crossed 12 independent homozygous driver lines with five independent homozygous effector lines to generate 60 different combinations (a crossing scheme presenting data on all 60 *trans*-heterozygous combinations is shown in Additional File 1). From each combination, we collected eggs to visualize the early expressed *tTA *and proapoptotic gene *hid*^*Ala*5 ^by *in situ *hybridizations. The lethal activity of each combination was checked by a second egg collection, which was counted for eggs and progeny. To describe the dimension of lethality we henceforth use the term 'complete lethality' for 100% lethality in laboratory experiments. The lethality was checked in this *trans*-heterozygous condition, since the heterozygous condition must suffice to induce lethality when homozygous males are crossed with WT flies in intended applications. All combinations that showed detectably lower or no progeny under these *trans*-heterozygous conditions were then inbred to generate homozygous (for both driver and effector construct) lethality lines (LLs). In addition four combinations with non-reduced progeny were made homozygous for comparison (see Additional file 1).

All LLs expressed *tTA *specifically during cellularization. However, due to the different P/Es as well as integration sites, the *tTA *expression strength varied and resulted in different expression strengths of *hid*^*Ala*5 ^(Figure 2 and Figure 3). This resulted in variable efficiencies of the lethality system. LLs deriving from the same driver line showed similar expression levels of *tTA*. The P/Es *sl1 *(0.4 kb; Figure 2 A1–3) and *99 *(Figure 2 C1–3) mediated only very weak expression of *tTA*, which subsequently could not induce detectable levels of *hid*^*Ala*5 ^expression. The longer P/E region of *sl2 *(1.9 kb) was able to drive *tTA *and, indirectly, *hid*^*Ala*5 ^(Figure 2 B1–6), but the level of *hid*^*Ala*5 ^expression was not high enough to drive complete lethality (Figure 2). With the P/E *CG2186 *we obtained a strong level of *tTA *expression during cellularization (Figure 2 D2), which started the expression of *hid*^*Ala*5 ^during the cellularization stage (Figure 2 D5) and led to complete pupal lethality of LL #68 (Figure 2).

Besides the finding that different P/Es or P/E regions act differently on *tTA *and the dependent *hid*^*Ala*5 ^expression, also the integration site of the driver construct could influence the *tTA *expression (Figure 3). Three independent lines, carrying the driver construct with the *sry α *P/E at different integration sites, expressed the *tTA *specifically but with different strengths during cellularization (Figure 3 A2–C2). In LL #65, a weak expression of *tTA *led to a late expression of *hid*^*Ala*5 ^during germ band retraction, which was not able to drive complete lethality (Figure 3). In contrast, the LLs #66 and #67 expressed *tTA *strongly during cellularization (Figure 3 B2, C2), which activated *hid*^*Ala*5 ^expression at the cellularization stage (Figure 3 B5, C5) and led to complete L1 larval lethality for LL #66 and complete embryonic lethality for LL #67 (Figure 3). Thus, a strong *tTA *expression seems to be important to start the *hid*^*Ala*5 ^expression early enough to cause complete embryonic lethality.

In addition, the effector constructs with different basal *D. melanogaster *promoters or different integrations of the same effector construct influence the levels of *hid*^*Ala*5 ^expression and lethality. The effector constructs *TREp-hid*^*Ala*5 ^and >*TREp-hid*^*Ala*5^>, carrying the *p*-basal promoter, were able to express *hid*^*Ala*5 ^in medfly after activation through the 12 independent driver lines, but did not cause complete lethality in 36 different combinations (Additional file 1). Interestingly, the effector construct *TREhs43-hid*^*Ala*5^, which carries the basal promoter (43 bp) of *D. melanogaster hsp70*, showed differences in the expression strength of *hid*^*Ala*5 ^depending on the integration site of the construct. In comparison with the larval or embryonic LLs #66 or #67, which are derived from the effector line *TREhs43-hid*^*Ala*5^*_*F1m2, the *hid*^*Ala*5 ^expression in LLs #29 and #72 deriving from *TREhs43-hid*^*Ala*5^*_*F1m1 started at later stages (Figure 3 D6, E6) and was not sufficient to drive complete lethality at the embryonic, larval, or pupal stage. Thus, we can show that the choice of E/Ps as well as the integration site of both the driver and the effector is crucial to set up a successful embryonic lethality system.

### Molecular and cytogenetic characterization of transgenic lines

**Figure 4:**
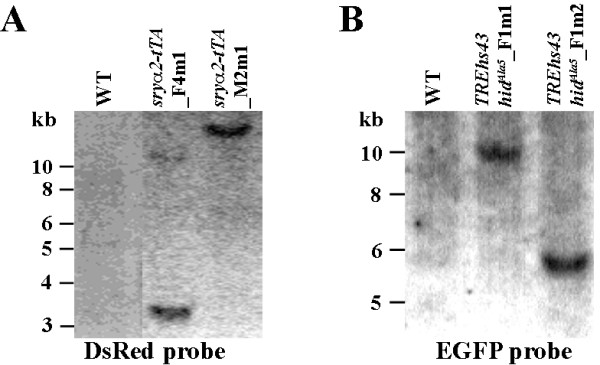
**Southern hybridizations**. *Bam*HI-digested genomic DNAs isolated from indicated medfly lines were hybridized with DsRed (A) or EGFP (B) probes, respectively (see *Methods*). Wildtype (WT) genomic DNA was used as a control for both. A single band in each lane indicates single integrations of the transgenes.

**Figure 5:**
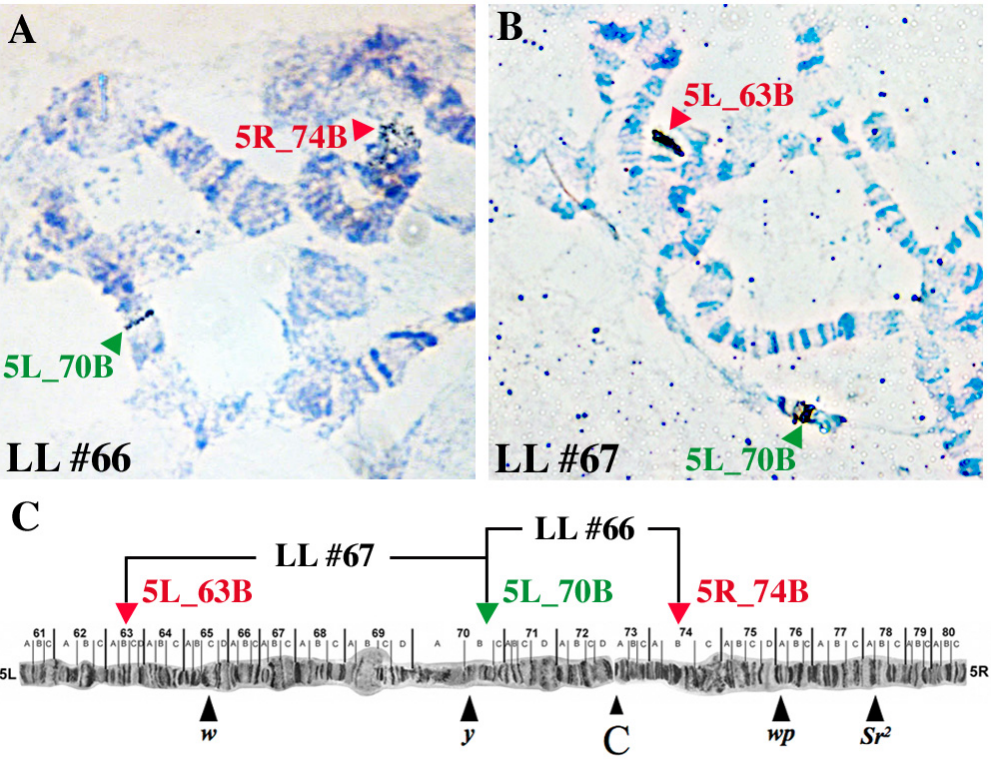
**Chromosomal localization of transgenes in embryonic LL #66 and #67**. An *in situ *hybridization on spread chromosomes from LL #66 (A) and LL #67 (B) is shown and the integrations are schematically represented in respect to other genetic markers (black arrowheads) on the fifth chromosome (C). The centromere is indicated as C. The integration site of the driver construct *sryα 2-tTA*_PUbDsRed was recognized at 5R_74B for LL #66 (A, red arrow) and at 5L_63B for LL #67 (B, red arrow). The effector construct *TREhs43-hid*^*Ala*5^_PUbEGFP was recognized at position 5L_70B (A-C, green arrows). The signals were assigned to the respective constructs by comparing the chromosomal locations of LL #66 and #67, deriving from the same effector but different driver lines.

Transgenic driver and effector lines were preliminarily screened for homozygous condition by fluorescence patterns and intensity. Southern blots indicated single copy integrations of driver and effector constructs of the lines *sryα 2-tTA*_F4m1, *sryα 2-tTA*_M2m1, *TREhs43-hid*^*Ala*5^_F1m1, and *TREhs43-hid*^*Ala*5^_F1m2 (Figure 4). Moreover, the correct *piggyBac*-mediated integrations at canonical *TTAA *target sites were verified by isolation of 5' and 3' insertion site sequences by inverse PCR (see Additional file 2). Therefore we know that differences in expression strength and functionality of the lethality system in different LLs are not a result of multiple insertions of the driver or effector constructs, but must be due to position effects. Furthermore, the integration sites of the driver and effector construct for LLs #66 and #67 were mapped by chromosome spreads. We found the driver and the effector of LL #66 or LL #67 located on chromosome 5 at the positions 74B and 70B or 63B and 70B, respectively (Figure 5). Thus, both LLs #66 and #67 have been results of recombinations. LL #66 was more difficult to establish than LL #67, corresponding to an expected lower recombination frequency between loci 74B and 70B than between loci 63B and 70B (Figure 5).

### Maternal suppressibility of lethality and its reversibility

Females feeding on adult food containing a suppressor, tetracycline (Tc) or doxycycline (Dox), should be able to transfer the suppressor molecules maternally into the oocytes. After egg laying, this maternally contributed suppressor should bind to the tTA protein produced during early embryogenesis, blocking the *trans*-activating potential of tTA and thus the switch of the lethality system. To identify the minimal concentrations of Tc needed to rear the LLs #29, #72, #66, #67, and #68, flies were bred on larval and adult media containing different concentrations of Tc. We defined the optimal Tc concentration in adult and larval medium for rearing as the lowest possible amount of Tc combined with the highest possible number of descendants. The LLs #72, #66, and #67 could be reared efficiently on adult medium containing 10 μg/ml Tc and LL #29 even on 1 μg/ml Tc. All LLs could be reared on larval medium containing 1 μg/ml Tc, except LL #68 (10 μg/ml Tc). LLs #66 and #67 were in addition tested to be efficiently rearable on larval medium containing only 0.1 μg/ml Tc or Dox. When reared on larval medium lacking Tc, all lines showed a reduction of progeny. Rearing LLs #66 and #67 on adult food containing 100 μg/ml Dox resulted in maternal suppression of the lethality, even without Tc or Dox in the larval food. Hatching and pupation rates were comparable to WT flies reared on adult and larval food without Dox. Thus, Dox is able to maternally suppress the embryonic lethality. However, eclosion rates were still reduced when Dox was used in adult food only (data not shown).

In addition all lines and WT showed delayed ovary development and 5–7 day postponed egg laying when reared on adult medium containing 100 μg/ml Tc and larval medium containing 300 μg/ml Tc. This indicates the importance of reducing the Tc concentrations to a minimum for the efficient rearing of medfly lines.

**Figure 6:**
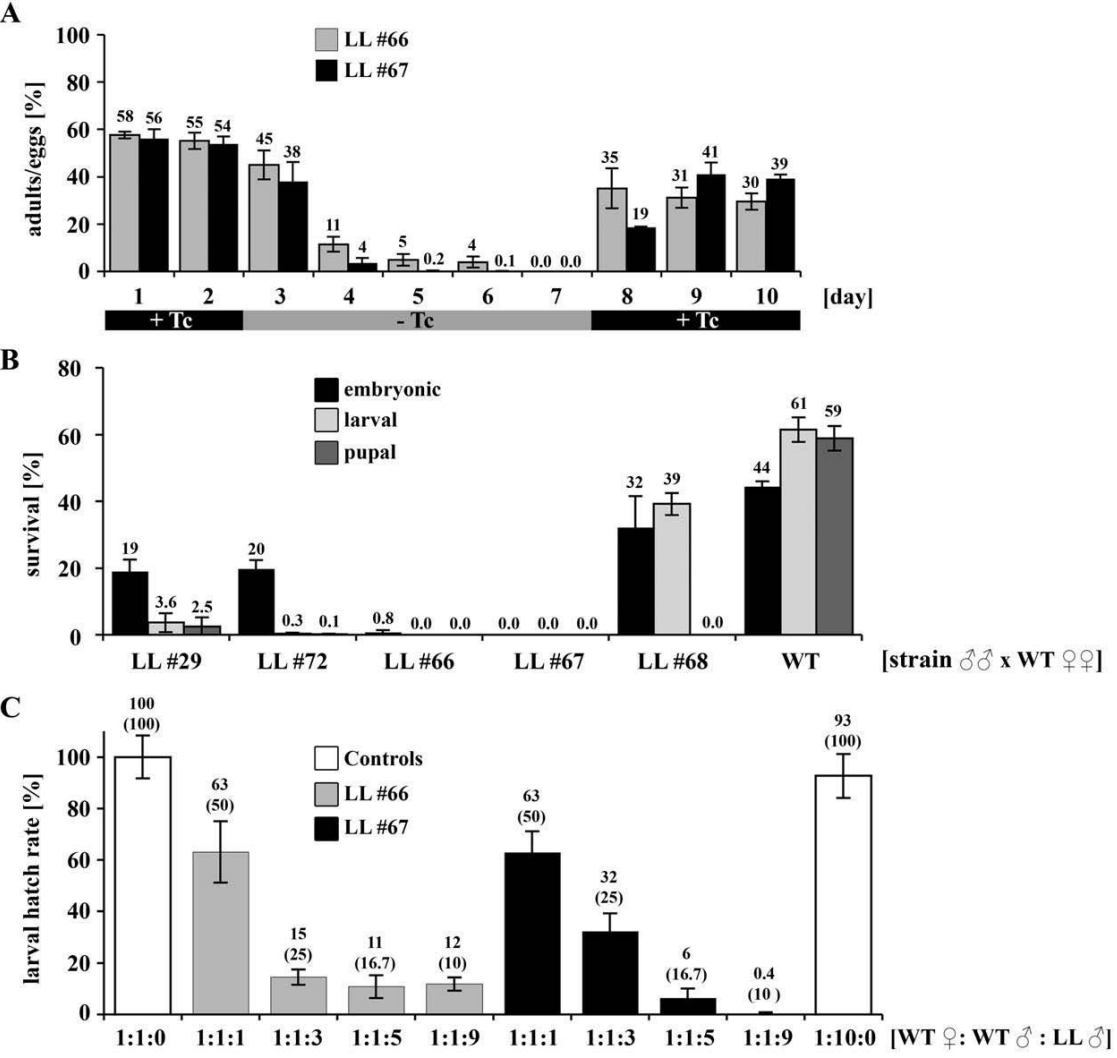
**Reversibility, efficiency, and competition tests**. (A) Reversible lethality: three-day-old flies from LLs #66 and #67 were reared on Tc-containing food (+Tc; 10 μg/ml) for two days, transferred to Tc-free medium (-Tc) for five days and transferred back to Tc-containing food for three days. Progeny of 24 h egg lay intervals were monitored (embryos from Tc-containing or Tc-free adult medium were reared on 1 μg/ml Tc-containing or Tc-free larval food, respectively). The ratio of adults to laid eggs is shown. For comparison, the ratio of eclosed adults to laid eggs in wildtype (WT) was in a range of 54%–74% under our rearing conditions (not shown). Two repetitions of the time series were performed. The SD of these two repetitions is indicated. Differences between repetitions are non-significant (ns), as shown by chi-test (Additional file 5). (B) Efficiency test: Shown are the combined data of four repetitions (see also Additional file 3). Total hatched L1 larvae 48 h after egg collection, total pupae, and total adults were counted and are shown in relation to the total number of eggs (total egg number: *n *(#29) = 1499; *n *(#72) = 4330; *n *(#66) = 2278; *n *(#67) = 2058; *n *(#68) = 1914; *n *(WT) = 2411). Due to difficulties in the larval count, the number of surviving larvae might be an under-representation. The SD of four repetitions is indicated. Differences between repetitions are ns, as shown by t-tests (Additional file 5). (C) Competition for virgin WT females: Numbers are normalized to the positive control (1:1:0). The expected larval hatch rate is indicated in brackets. The SD of two repetitions (each independent repetition consisting of the six egg collections) is indicated. Differences between repetitions are ns, as shown by t-tests (Additional file 5).

To test for reversible sterility of lines #66 and #67, adults were reared on Tc-containing medium (10 μg/ml) for two days (Figure 6A). After transfer to Tc-free medium the rate of progeny decreased in five days to 0%. The sterility could be reversed by retransfer of the adults to Tc-containing medium. The reduced rate of progeny after this procedure could be due to a slight irreversible effect of the lethal system or to the advanced age of flies, as shown in other studies [16].

### Efficiency and competition tests

During medfly SIT programs, irradiation sterilized males are released into affected areas and mate with WT females, which leads to infertile matings. Ideally all progeny die as embryos to exclude damage to fruits from larval feeding. To show the efficacy and time point of lethality for the newly generated LLs, transgenic males (homozygous for driver and effector) from LLs #29, #72, #66, #67, #68, or WT were crossed with WT females, respectively (Figure 6B and Additional file 3). For the LLs #29 and #72, about 20% of the eggs survived to become L1 larvae, whereas pupae and adult progeny were highly reduced. Crossings with males from LLs #66, #67, and #68 showed complete pupal lethality but varying larval and embryonic lethality. Only 0.8% of the laid eggs from the LL #66-crossing hatched and all of those died during L1 larval stage. The LL #67-crossing showed the desired complete embryonic lethality.

**Figure 7:**
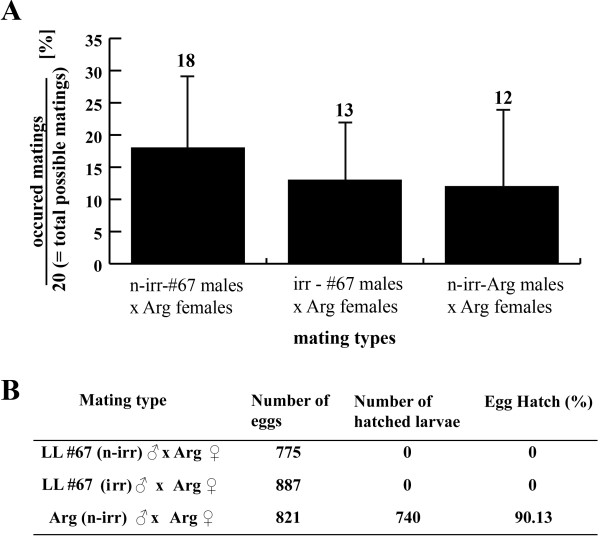
**Mating competitiveness of LL #67 in field cage tests**. To test the competitiveness of the embryonic LL #67, 20 non-irradiated and 20 irradiated males from LL #67 (120 Gy) competed with 20 non-irradiated wildtype (WT) Argentinean (Arg) males for mating with 20 WT Arg females in a field cage [17]. The males were marked with different colored water-based paints. Mating couples were taken out of the cage and the type of mating couple was recorded. Twelve replications were carried out. (A) The proportion of matings of each mating type was calculated by dividing the number of the occurred matings by the number of total possible matings (limited by the number of Arg females, *n *= 20). The proportion of matings was 18 ± 11% for non-irradiated LL #67 males, 13 ± 9% for irradiated LL #67 males, and 12 ± 12% for non-irradiated Arg males. The proportion of total matings over all 12 replications was 43 ± 5%, indicating an acceptable degree of sexual activity during the test period. Running a conventional ANOVA, no statistical differences (*F *= 1.62; *P *= 0.171) can be found between the different matings that occurred. The tests thus showed that non-irradiated and irradiated LL #67 males were at least as, if not more, competitive as WT non-irradiated Arg males. (B) Eggs and hatched larvae from each mating type were recorded and the egg hatch is shown. All matings of LL #67 males (regardless of whether non-irradiated or irradiated) to WT Arg females led to complete embryonic lethality. In comparison with the complete lethality of LL #67 (descending from *EgII*) with or without irradiation, previous sterility tests with irradiated WT *EgII *males (100 Gy) showed an egg hatch of 1.2% [35]. In addition, radiation-induced sterility has been shown to be indirectly correlated to the competitiveness of the flies [4].

An ideal line for releasing purposes should be embryonic lethal, but should also be competitive. We therefore did competition tests with LLs #66 and #67 (Figure 6C). WT females were crossed with WT males and transgenic males in different ratios (1:1:1, 1:1:3, 1:1:5, and 1:1:9; these numbers represent the ratio of WT females: WT males: transgenic males). The reduction of progeny compared with WT-only controls showed that both lines are highly competitive. Remarkable is the higher fertilization success of LL #67 males compared with WT males starting from ratio 1:1:5. For the ratio 1:1:9 an overall progeny rate of only 0.4% was measured. At the same time a WT control at ratio 1:10:0 gave only little reduction of overall progeny (Figure 6C). Thus, transgenic males from LLs #66 and #67 performed in laboratory competition tests comparably or even better than WT males. Progeny from all competition tests were identified as non-transgenic individuals by fluorescent microscopy, which additionally indicated the complete lethality of LLs #66 and #67. Interestingly, all lines deriving from the effector line *TREhs43-hid*^*Ala*5^_F1m2 (#66, #67, and #68) partially lacked anterior orbital bristles, which did not obviously interfere with the mating success of these transgenic males. In addition to laboratory tests, field cage tests [17] with LL #67 males showed a comparable or even better competitiveness than WT (Figure 7).

## Discussion

In this study we describe the first transgenic embryonic lethality system for the insect pest *C. capitata *that causes complete reproductive sterility without the need of radiation. The use of a newly isolated early embryonic P/E of medfly makes possible a conditional embryonic lethality without larval hatching. The described embryonic lethality system has the advantage of eliminating the radiation process, a possible release of insects at any life-cycle stage, and an expected fitness benefit of transgenic males over radiated males. Other transgenic lethality systems without the need of radiation, such as RIDL [18], reduce the eclosion rate of flies, but as larvae are still produced, larval damage to targeted food would still be present and transgenes would be transferred into the wild population due to pupal survival. These transgenes would carry along transgenesis markers that will also interfere with effective monitoring of SIT programs. In contrast, the newly developed embryonically lethal medfly strains produce reproductively sterile males, which would mate to WT females after release and their progeny would die during embryogenesis. This would prevent larval hatching and the introgression of transgenes into WT medfly populations.

In the presented embryonic lethality system, the tTA expression mediated by cellularization-specific P/Es not only depends on the P/E itself but also on the integration sites in the genome. These differences in expression led to a variety of lethality levels in the 60 tested transgene combinations. Interestingly, the basal promoters of the *P *element (*p*) and *hsp70 *(*hs43*) from *D. melanogaster *were both able to drive the lethal factor *hid*^*Ala*5 ^after *TRE*-mediated activation by tTA in *C. capitata*. However, effectors containing the *p *basal promoter could not promote complete lethality, whereas systems with effectors containing *hs43*, which gave no functional transgenes in *D. melanogaster *[6], caused complete lethality in medfly at different stages of development. This demonstrates that even a molecularly well-constructed system is highly dependent on the specific P/Es used and on the integration sites of the transgenes.

The additional finding that both constructs of the lethal and competitive LLs #66 and #67 are located on chromosome 5 has several advantages. First, these lines can be combined with different well-established systems located on chromosome 5: e.g. a phenotypic marker system [19] or part of a genetic sexing system, as in genetic sexing strain (GSS) *Vienna-8 *[20]. The advantage of having different systems on chromosome 5 is a simplified quality control during rearing procedures. Second, the embryonic lethality line brings about two fluorescent markers (DsRed and EGFP), which are not only helpful during quality control but could also help in monitoring processes. Third, our constructs introduced *attP *sequences, which will allow to site-specifically modify these competitive embryonic LLs by using the integrase system from phage *phiC31 *[14]. Possible applications will be the deletion of *piggyBac *ends [21] to further increase the safety of transgenes or insertion of recently developed sperm markers for improved monitoring [16].

To suppress lethality during mass rearing, Tc or Dox can be used as a supplement in the food. In laboratory assays, we were able to reduce the Tc concentrations in rearing media to 0.1 μg/ml at larval and to a minimum of 10 μg/ml at adult stage. Using these Tc concentrations for rearing, we could not detect any *hid*^*Ala*5 ^expression during embryogenesis. Dox at 100 μg/ml in the adult food was sufficient to maternally suppress the embryonic lethality without the need for Dox in the larval food. However, eclosion rates were reduced when Dox was used in the adult food only, which suggests the need for some Tc or Dox in the larval food. In addition, we observed a delayed ovary development and postponed egg laying when 300 μg/ml Tc was used in larval medium in combination with 100 μg/ml used in adult medium. The described Tc concentrations for the embryonic lethality system are 10–1000 times lower than published Tc concentrations needed to suppress lethality of other medfly lethality systems [18,22]. Compared with these other transgenic systems, the much lower Tc concentrations would reduce costs of mass rearing.

Both LLs #66 and #67 show high competitiveness to WT flies in laboratory tests and LL #67 also in field cage tests. These transgenic LLs can now be used to evaluate the fitness costs of transgenic lethality compared with radiation-based sterility. The current 100-fold inundation of affected areas with radiation-sterilized males could be reduced to lower amounts with more competitive LLs and at the same time embryonic lethality could be maintained. We show that the embryonic LL #67 is a '100% sterility'-system. Such systems are required for preventional SIT programs in California or Florida [23] and are desired for every other pest management program. Using the embryonic lethal strains would eliminate the problem of introducing transgenes into wild medfly populations by surviving flies as described for the conditional lethality system in Gong et al. [18]. In addition, the use of the embryonic lethality system without the need of radiation can increase the safety of the mass-rearing process during operational SIT programs, since accidental releases would not lead to infestations of the environment [24] and possible risks coming from isotopic sources could be eliminated for workers and the environment.

## Conclusion

The first successful transfer of the *Drosophila *proof-of-principle embryonic lethality system to an agricultural pest, the medfly *C. capitata*, represents a straightforward approach that can be applied to further pest insects. We show that complete embryonic lethality in *C. capitata *is possible and that the responsible transgenes do not reduce competitiveness. The 100% embryonic lethal system is proposed as an alternative to reproductive sterility achieved by radiation for pest management programs based on the sterile insect technique.

## Methods

### Medfly samples

WT and transgenic medfly lines were maintained under standard rearing conditions [25]. The WT strain *Egypt-II *(*EgII*), which has been reared in laboratories for more than 25 years, was obtained from the FAO/IAEA Agriculture and Biotechnology Laboratory (Entomology Unit, Seibersdorf, Austria). Laboratory tests for efficiency, competition, and reversibility of the lethality system were performed in 15 × 15 × 20 cm acrylic cages. The Argentinean WT strain (Arg; from Mendoza, Argentina) was reared for about 20 generations under relaxed artificial rearing conditions (Entomology Unit, Seibersdorf, Austria) and then used for the field cage tests.

### Isolation of cellularization-specifically-expressed genes

The Clontech PCR-Select cDNA Subtraction Kit (BD Biosciences, Heidelberg) was used to isolate fragments of the following genes expressed specifically during cellularization as described [9]: *C.c.-slam*, *C.c.-sub2_99*, *C.c.-CG2186, C.c.-sub2_63*, and *C.c.-sub2_65*. An EST fragment of the medfly cellularization gene *serendipity α *(*C.c.-sry α*) was received from Dr. Ludvik Gomulski, Pavia. By RACE, 5' and 3' ends of cellularization-specific genes were isolated using the BD SMART RACE cDNA Amplification Kit (BD Biosciences, Heidelberg) and gene-specific primers. Complete cDNA sequences are deposited to GenBank [accession numbers: FJ460697 (*Cc_slam*), FJ460699 (*Cc_sub2–99*), FJ460701 (*Cc_CG2186*), FJ460705 (*Cc_sub2–63*), FJ460706 (*Cc_sub2–65*), FJ460703 (*Cc_sry α*)].

### Inverse PCR

Inverse PCR was performed to get the 5' regions of genes specifically expressed during cellularization: 1.5 μg of medfly WT genomic DNA was digested for 24 h; restriction fragments were precipitated and self-ligated in a volume of 500 μl at 16°C for 24 h; PCR was performed on circularized fragments by using primer sequences (for a complete list of primers see Additional file 4) in opposite orientation within the 5' UTR or ORF of the genes. First, PCRs (1 min at 95°C; 6 cycles of 30 sec at 94°C, 45 sec at 66°C (-2°C each cycle), 6 min at 68°C; 25 cycles of 30 sec at 94°C, 45 sec at 54°C, 6 min at 68°C; and 6 min at 68°C) for *C.c.-slam, C.c.-sub2_99, C.c.-CG2186, C.c.-sry α *or *C.c.-sub2_63 *were performed on *Fsp*BI, *Nde*I, *Cvi*AII, *Pvu*I *or Acl*I cut genomic DNA with the primer pairs mfs-77/-79, mfs-85/-108, mfs-170/-172, mfs-159/-161 or mfs-83/-104, respectively, using BD Advantage 2 PCR (BD Biosciences, Heidelberg). Second, the obtained PCR products were diluted 1:50 with ddH_2_0 and nested PCRs with primer pairs mfs-78/-80 (*C.c.-slam*), mfs-160/-162 (*C.c.-sryα*) or mfs-171/-173 (*C.c.-CG2186*) were performed (1 min at 95°C; 22 cycles of 30 sec at 94°C, 45 sec at 54°C, 6 min at 68°C; and 6 min at 68°C) using 5 μl of the dilution and the BD Advantage 2 PCR Kit (BD Biosciences, Heidelberg). PCR products from first (*C.c.-sub2_99 *and *C.c.-sub2_63*) and nested PCRs (*C.c.-slam, C.c.-sry α *and *C.c.-CG2186*) were cloned into pCRII vectors (Invitrogen, Karlsruhe) and sequenced. Upstream sequences are deposited to GenBank [accession numbers: FJ460696 (*upstream_Cc_slam*), FJ460698 (*upstream_Cc_sub2–99*), FJ460700 (*upstream_Cc_CG2186*), FJ460704 (*upstream_Cc_sub2–63*), FJ460702 (*upstream_Cc_sryα*)].

To localize the integration sites of *piggyBac *vectors, inverse PCR was performed with primers and protocols as described [26]. Sequences flanking *piggyBac *insertions are shown in the Additional file 2.

### Two-step cloning procedure

Generally we compose our constructs in the cloning shuttle vector pSLfa1180fa. From the shuttle vectors the constructs can be easily placed in transformation vectors which carry *Fse*I and *Asc*I sites (*fa*-sites [27]).

### Shuttle vectors

The pSL*af_attP-sl2-tTA_af *(#1231), pSL*af_attP-63-tTA_af *(#1232), pSL*af_attP-99-tTA_af *(#1234), pSL*af_attP-sryα 2-tTA_af *(#1236), and pSL*af_attP-CG2186-tTA_af *(#1237) carry a 52 bp *attP *site [28]. #1231, #1232, or #1234 was created by ligating annealed *attP *primers (mfs-201/-202) in the *Eco*RI cut pSL*af_sl2-tTA_af *(#1210), pSL*af_63-tTA_af *(#1211) or pSL*af_99-tTA_af *(#1212), respectively. #1236 or #1237 was created by ligating annealed *attP *primers (mfs-203/-204) in the *Nco*I cut pSL*af_sryα 2-tTA_af *(#1225) or pSL*af_CG2186-tTA_af *(#1226), respectively.

#1210, #1211 or #1212 was created by ligating the *Eco*RI-*Xba*I cut *sl2 *fragment (a 1.9 kb 5'-region of the gene *C.c.-slam*), the *Eco*RI-*Eco*31I cut 63 fragment (a 1.2 kb 5'-region of the gene *C.c.-sub2_63*) or the *Eco*RI-*Xba*I cut *99 *fragment (a 0.7 kb 5'-region of the gene *C.c.-sub2_99*), amplified by PCR on genomic DNA with primer pairs mfs-141/-113, mfs-142/-143 or mfs-131/-133, in the *Eco*RI-*Xba*I cut pSL*af_tTA_af *(#1215), respectively. #1225 or #1226 was created by cloning the *Nco*I-*Xba*I cut *sryα 2 *fragment (a 1.6 kb 5'-region of the gene *C.c.-sry α*) or the *Nco*I-*Eco*31I cut *CG2186 *fragment (a 1.2 kb 5'-region of the gene *C.c.-CG2186*), amplified with primer pair mfs-189/-188 or mfs-190/-191, in the *Nco*I-*Xba*I cut #1215, respectively. #1215 was generated by cloning a 1.5 kb *Xba*I-*Hind*III cut *tTA-SV40 *fragment from pTetOff (Clontech, CA) in the *Xba*I-*Hind*III cut pSLfa1180fa [27].

### Transformation vectors

The driver construct p*Bac{sl1-tTA_PUb-DsRed} *(*sl1-tTA*) was generated by ligating the *Bgl*II/*Xba*I cut *sl1 *(a 0.4 kb 5'-region of the gene *C.c.-slam *amplified with primer pair mfs-112/-113 from genomic DNA) and the *Xba*I/*Bgl*II cut *tTA-SV40 *(a 1.5 kb region amplified with primer pair mfs-110/-111 from pTetOff) in the *Bgl*II site of pB [PUbDsRed1] [29].

The driver constructs p*Bac{f_attP-sl2-tTA_a_PUb-DsRed} *(*sl2-tTA*), p*Bac{f_attP-63-tTA_a_PUb-DsRed} *(*63-tTA*), p*Bac{f_attP-99-tTA_a_PUb-DsRed} *(*99-tTA*), p*Bac{f_attP-sryα 2-tTA_a_PUb-DsRed} *(*sryα 2-tTA*) or p*Bac{f_attP-CG2186-tTA_a_PUb-DsRed} *(*CG2186-tTA*) were generated by ligating the *Fse*I-*Asc*I fragment *attP-sl2-tTA, attP-63-tTA, attP-99-tTA, attP-sryα 2-tTA *or *attP-CG2186-tTA *from #1231, #1232, #1234, #1236 or #1237 in the *Fse*I-*Asc*I cut p*Bac{fa_PUb-DsRed} *(#1200 [16]), respectively.

The effector constructs p*Bac{fa_attP_f_TREp-hid*^*Ala*5^*_a_PUb-EGFP} *(*TREp-hid*^*Ala*5^) or p*Bac{fa_attP_f_TREhs43-hid*^*Ala*5^*_a_PUb-EGFP} *(*TREhs43-hid*^*Ala*5^) were generated by cloning the hybridized primers mfs-211/-212 in the *Xma*JI site of p*Bac{faf_TREp-hid*^*Ala*5^*_a_PUb-EGFP} *(#1207) or p*Bac{faf_TREhs43-hid*^*Ala*5^*_a_PUb-EGFP} *(#1208), respectively. #1207 or #1208 were created by ligating the *Asc*I fragments *TREp-hid*^*Ala*5 ^(5.0 kb) or *TREhs43-hid*^*Ala*5 ^(4.9 kb) from pSLfa_*TREp-hid*^*Ala*5^_fa or pSLfa_*TREhs43-hid*^*Ala*5^_fa [6] in the *Asc*I site of p*Bac{fa_PUb-EGFP} *#1201 [16], respectively. The effector construct p*Bac{>fa_attP_f_TREp-hid*^*Ala*5^*_a>_PUb-EGFP} *(>*TREp-hid*^*Ala*5^>) was generated by ligating the *Asc*I-fragment *attP_f_TREp-hid*^*Ala*5 ^from *TREp-hid*^*Ala*5 ^in the *Asc*I-site of p*Bac{>fa>_PUb-EGFP} *[16].

### Germline transformation

Germline transformation experiments were performed by microinjection of *piggyBac *constructs (500 ng/μl) together with the p*hspBac *transposase helper plasmid (200 ng/μl) [30] into WT embryos as described by Handler and James [31] with the following exceptions: injected eggs were covered with Voltalef 10S oil (Lehmann & Voss, Hamburg, Germany), placed at 28°C in parafilm closed Petri dishes with watered Whatman paper in the lid; neither eggs, larvae nor pupae were heat shocked.

The vectors *sl1-tTA*, *sl2-tTA*, *99-tTA*, *sryα 2-tTA*, *CG2186-tTA*, *TREp-hid*^*Ala*5^, *TREhs43-hid*^*Ala*5^, or >*TREp-hid*^*Ala*5^> were injected into 600 embryos of which 260, 140, 160, 54, 83, 28, 63, or 52 survived to adulthood, respectively. Four female crossings (two to 25 G_0_ females crossed with 15 WT males; F1–F4) and four male crossings (two to 25 G_0 _males crossed with 15 WT females; M1–M4) were set up for each construct. G1 progeny were screened by epifluorescence for the expression of the *PUb-DsRed *or *PUb-EGFP*. Fluorescent progeny with different red or green patterns were backcrossed twice to WT to recognize possible multi-insertions and brought to homozygous conditions by inbreeding and checking fluorescence intensity. For screening of flies we used the fluorescence stereomicroscope Leica MZ16 FA with the filters DsRedwide (Ext. 546/12; Emm. 605/75) and EYFP (Ext. 500/20; Emm. 535/30).

To generate LLs, we crossed 12 homozygous driver lines and five homozygous effector lines in all possible combinations. We inbred those heterozygous combinations, which produced detectably lower or no progeny, screened the progeny by fluorescent intensity for homozygous individuals, and subsequently inbred these homozygous individuals to generate LLs homozygous for driver and effector construct.

### Southern hybridization

Genomic DNA (~3–10 μg) from adult flies of different transgenic lines and the WT strain was digested with *Bam*HI (Roche, Mannheim) and separated on 1% agarose gels. DNA was transferred to nylon membranes (Hybond-N^+^; GE Healthcare/Amersham, Little Chalfont) and immobilized by UV irradiation. Probe labeling and membrane hybridizations were performed according to the AlkPhos Direct kit (GE Healthcare, Little Chalfont). Signal detection was performed using CDP-star (GE Healthcare, Little Chalfont) followed by exposure for approximately 30 min on Kodak Biomax ML film.

The two probes for detecting DsRed or EGFP were amplified by PCR (2 min at 94°C; 30 cycles of 30 sec at 94°C, 30 sec at 53°C, 1 min at 72°C; 5 min at 72°C) from the constructs #1200 or #1201 with the primers mfs-333 and mfs-334 or mfs-335 and mfs-336, respectively.

### *In situ *hybridization

WMISH with RNA probes to embryos were performed as described [32]. RNA antisense probes were prepared by *in vitro *transcription with the DIG-RNA-Labeling Kit (Roche, Mannheim) from pCRII vectors (Invitrogen, Karlsruhe) containing subtraction cDNA fragments (p_*slam*, p_*99*, p_*CG2186*, p_*63*, p_*65*), an EST fragment (p_*sryα*), and the plasmids pBSK-*hid*^*Ala*5 ^or pBSK-*tTA *[6]. By PCR using the primer pair mfs-41/-42, cDNA fragments were amplified and transcribed with Sp6 polymerase. The plasmids p*BSK-hid*^*Ala*5 ^or p*BSK*-*tTA *were linearized with *Cla*I or *Eco*RI and transcribed with T3 or T7 RNA polymerase, respectively.

### Chromosome spreads

Chromosome *in situ *hybridizations were performed with slight modifications as described [33]. Instead of horseradish peroxidase, the Biotin/Avidin system VECTASTAIN Elite ABC was used (Vector laboratories, Peterborough). Hybridization sites were identified and photographed using 60× oil objectives (Olympus phase contrast microscope) with reference to medfly salivary gland chromosome maps [34]. Squash preparations of salivary gland polytene chromosomes were made as described [33]. A DNA-probe recognizing *piggyBac *insertions was prepared by PCR on genomic DNA from flies carrying a DsRed-marked *piggyBac *insertion [29] with the primers DsRed_F and DsRed_R (1813 bp) using the Biotin High-Prime kit (Roche Diagnostics, Mannheim).

### Efficiency test of induced lethality

Tc-free adult and larval food was used. In four independent repetitions, virgin WT females were crossed directly after eclosion with homozygous males from LLs #29, #72, #66, #67, #68, or WT, respectively. Four days later a 24 h egg collection was taken from each crossing. For statistical analyses on the reversibility, efficiency, and competition tests please see Additional file 5.

### Laboratory mating competitive tests

15 WT females and 15 WT males were placed together with different numbers of males of LLs #66 or #67 (15 (1:1:1) – 135 (1:1:9)). For control matings, 15 virgin WT females were crossed with either 15 WT males (1:1:0) or 150 WT males (1:10:0). Two independent crossings were performed for each ratio. Six 24 h egg collections were obtained from each crossing and the number of adult progeny was recorded. Adult progeny were verified by fluorescence light microscopy as WT or transgenic offspring.

### Field cage tests for mating competitiveness

Males from LL #67 (non-irradiated or irradiated with 120 Gy 48 hours before adult emergence) were competed against non-irradiated WT Argentinean males for mating with Argentinean WT females in a field cage (field cage size (W-D-H): 3 m-3 m-2.3 m; [17]). Pupae from the different strains/treatment were placed in emergence cages, and every 24 h adults were removed, sorted by sex, and placed in cages with adult food (3:1, sugar:hydrolyzed yeast) and water for 6 days. Two days before the tests, flies were marked with a dot of water-based paint on the thorax (DEKA^®^, Unterhaching, Germany). In each field cage, three potted *Citrus aurantius *trees, 1.6 m in height with 1.5 m diameter canopy, were used as a mating arena. To follow the quarantine protocol, tests were performed in a greenhouse with controlled temperature (24–26°C) and humidity (60–80%). On the day of the test, 20 sexually mature non-irradiated Argentinean males, 20 non-irradiated, and 20 irradiated males from line LL #67 were released into the cage around 08:30. Approximately 20 min later, 20 virgin and sexually mature Argentinean females were released in the cage. Tests lasted 3 hours. Mating pairs were collected as they formed by allowing the pair to walk into a small vial. The type of mating couple was recorded and the proportion of mating was calculated for each mating type (see Figure 7). After the couples separated, the males and females were identified and the mated females were grouped together depending on the type of mated male and transferred to small egging cages. Eggs were collected for five consecutive days and transferred to small Petri dishes with moist black filter paper. After four days of incubation, hatched larvae and un-hatched eggs were counted to determine the egg hatch for each mating type. Twelve replications of this test were carried out.

### Optimization of suppressor concentrations for rearing

Starting from larval and adult media Tc-concentrations of 100 μg/ml, the minimal Tc concentrations for the LLs #29, #66, #72, #67, and #68 were tested with WT as a control. First, flies were reared on adult medium containing 100 μg/ml Tc and eggs were collected on larval medium containing 0, 1, 3, 10, 30, 100, or 300 μg/ml Tc. Hatching, pupation and eclosion rates were recorded. Second, the adult medium Tc concentrations (1, 3, 10, 30, 100, or 300 μg/ml) were tested over three generations by using the optimized larval media concentrations (1 μg/ml for LLs #29, #66, #72, and #67; 10 μg/ml for LL #68) in between the adult stages.

Additionally, LL #66 and LL #67 were reared on adult media containing 10 μg/ml Tc and eggs were collected on larval medium containing 0.1, 0.3, and 1 μg/ml Tc or Dox. Hatching, pupation, eclosion rates, and viability of progeny from the eclosed flies were recorded.

Moreover, freshly eclosed flies from the LLs #66 and #67 were placed on adult food containing 100 μg/ml Dox. Four and six days after eclosion an egg collection was taken and the eggs reared on Dox-free larval medium. Hatching, pupation and eclosion rates were recorded and compared with data from WT (*EgII*) flies reared on Dox-free adult and larval food.

## Authors' contributions

MFS and EAW designed research; MFS, CC, and AZ performed research; MFS, CC, GF, and EAW analyzed data; and MFS and EAW wrote the paper.

## Supplementary Material

Additional file 1 **Crossing scheme of medfly lines carrying driver and effector constructs** Click here for file

Additional file 2 **Sequences flanking *piggyBac *insertions** Click here for file

Additional file 3 **Raw data of the efficiency test** Click here for file

Additional file 4 **Primer sequences shown in 5' to 3' orientation** Click here for file

Additional file 5 **Statistical analysis** Click here for file
