# Supplementary material for: Conditional embryonic lethality to improve the sterile insect technique in Ceratitis capitata (Diptera: Tephritidae)
Source: BMC Biol. 2009 Jan 27;7:4. doi: 10.1186/1741-7007-7-4 (PMC2662800; doi:10.1186/1741-7007-7-4)
Supplement: Additional file 1 — Crossing scheme of medfly lines carrying driver and effector constructs [file 1741-7007-7-4-S1.pdf]

Additional File 1. Crossing scheme of medfly lines carrying driver and effector constructs.

tTA is expressed in all the lines, but at variant levels. Detected hid^{Ala5} expression in progeny of the combinations is indicated with (H). Transheterozygous combinations with detectible reduction of progeny are marked in green. These five combinations and in addition one representative of each other driver construct crossed to the effector construct *TREhs43-hid*^{Ala5} F1m2 (indicated in bold) were choosen to generate homozygous lethality lines (LLs).

	*sl1-tTA_* F1f1	*sl1-tTA_* M6m1_HTA	*sl1-tTA_* F1f1_ETLA	*sl2-tTA_* F3m1_HTLA	*sl2-tTA_* F3m1_T	*99-tTA_* F2m1	*99-tTA_* M1f1	*99-tTA_* M4m1	*srya2-tTA_* F2m1	*srya2-tTA_* F4m1	*srya2-tTA_* M2m1	*CG2186-tTA_* F1m1	<= Homozygous driver lines
TREp-hid^{Ala5}_ F4m1	#55	#56	#34	#10 (H)	#11 (H)	#12	#13	#14	#15	#16 (H)	#17 (H)	#18 (H)	
TREp-hid^{Ala5}_ M4m1	#53	#54	#33	#01	#02	#03	#04	#05	#06	#07	#08 (H)	#09 (H)	
TREhs43-hid^{Ala5}_ F1m1	#59	#60	#36	#39 (H)	#43 (H)	#40	#41	#42	#28	#29 (H)	#72 (H)	#64	
TREhs43-hid^{Ala5}_ F1m2	#61	**#62**	#63	**#48** (H)	#49 (H)	#50	#51	**#52**	**#65**	#66 (H)	#67 (H)	#68 (H)	
>TREp-hid^{Ala5}*>*_ F1m1	#57	#58	#35	#19 (H)	#20	#21	#22	#23	#24	#25 (H)	#26 (H)	#27 (H)	

Homozygous effector lines =>
