# Supplementary material for: Conditional embryonic lethality to improve the sterile insect technique in Ceratitis capitata (Diptera: Tephritidae)
Source: BMC Biol. 2009 Jan 27;7:4. doi: 10.1186/1741-7007-7-4 (PMC2662800; doi:10.1186/1741-7007-7-4)
Supplement: Additional file 2 — Sequences flanking piggyBac insertions [file 1741-7007-7-4-S2.pdf]

Additional File 2

Sequences flanking *piggyBac* insertions. Sequences are shown in 5´ to 3´ orientation in respect to the *piggyBac* insertion. Duplicated integration sites (**ttaa**) indicated in bold, enzyme restriction sites underlined (ggcc = *Hae*III; ccgg = *Msp*I).

*sryα2-tTA*_F4m1

ccggaaacacaaattaaaacacttcccgatgcataaaacataatgtcaaaagcaaaatttccgctgctttatccgac
aaaatcaaataaattatcaacaatgtttatttacccaaagcacattatttccacaagcctttcagtgcggcttctaa
ttttactttctagatatttttcacaaaatatattttgcactaattttggaaagacgaaatctacgaataaaaacttc
ttcatcatatttctgaaagaaaatatttgtatacggcacgatctttccctataaatgcgcctctgaatcaacgcagc
gagtgcagtgcgttccatattttccataatgaaatttgtt**ttaa**…***piggyBac***…**ttaa**agccaacatccgg

*sryα2-tTA*_M2m1

ccggatatttaagtgataatcttttaaaccgtatctaaatacatattgtataaatataaataaattttgttactaat
ttattta**ttaa**…***piggyBac***…**ttaa**tgttcattttctccgttaaaaaatctctaattacagttcttgcacgcctacg
tttgatacttcctttgtaaacgtgtgactaataaaattcgccatcacgacgcagtacgcacttttaaccgttaaaca
cattatttgtgtctacaaggtgaagaaaaaattcatcgaatcagttaataatacataaaatactcgctaatcgccga
cattcagcagatattggtggaacaaacgcttgccaacgatcggacactagtcacagccgcaaccgcatacgccgtgt
agtaacgtccattctatcaaaccaacgtcgctttagccaaaccaacacccacccacacacacacactcacaatgccc
gttcaatctcccatcgcgattagcaatcgtgccattgaacatatcgatgatgtcgatcccttgaaatatcatggcca
ttgggaaccagttggtgttgcacgcgtacaaaatatccgg

TREhs43-hid^{Ala5}_F1m1

ggcctcctcaacagcttttctgtttgatacaacaaatctgtagagtcaaccttgagtaggggctgcagtagccaaga
agttagcagtactattatacagaggggttgactggaccaagatctgttcggcactcggcagagaatgtctaagaaat
cattgtgtaataattgggatatattttttatcccagaattccgtattccatttgttagatttcctcacgtttaaaaag
ctcataaagctcataacttcaactgtctcagcaacaaggcttcagaactaaggtatttgttagtttcaactttacgc
cgcacatcgatataattaatcgtgcaaattgcgttattgatttgttgtcctcaattcttatgctccgaacaaagtaa
attctaaacaaacccaacttcagttaaacattcaacaaataaaagttacgttgggggaaatttatttatatatacga
tattggcatatttaaagagggaccgttggaagagtaaaagcgtccctcatttttacaatt**ttaa**…***piggyBac***…**ttaa**
gagaggatatagaacatataaatccgagcgtatttccaacataattgtctcaaaagtttggatgtcttgaaactgag
ttcagaagagtgtcgtcagaaagggcttaacagtagatcggactcgtagctttcagtaaccttgcttaagattagtc
atggagattaggaggtccgggctactagcagtttcaatgaatttcggttttagtaatggttgttgtcttgtggaact
cactgaaggctgtttataacttacattggcc

TREhs43-hid^{Ala5}_F1m2

ggccaagtctggactgtaaggttggtgattaaaaatcgctcttatattgtcaacttatagaaacatatttttcttta
tgatcaatcaataatattctttccgagttccaaacgaccgtttgccatgagtttaccagctgactgtgaaatcttaa
accgaaaattgcactcatagact**ttaa**…***piggyBac***…**ttaa**aaatgtttgtatcggatacaccaagcgcttctgtaa
gttgtctcttctttggtggacgtcgttcggggacaatttttttctacctttggacaaattctcgcgtgatcaaaat
aaagagcaataaaaaactctctgctctactcacgttcgattagtgtttcaagtctttggagaagagtgatggcagaa
aggtacttgtcgagctggagagtatagttttgtcatctacaacggcatccaggtgttgatagagtgccttttttccat
tccaaacgttctggtatgcattcctgtatgaagatggcacctttcaccaactcattaatatcagttgtgcccgaaat
gagcgcgaattgttggttagcactgagtttgccacgcagtaagcccttcgattccaagcttttcagctccttttctg
tctcacgtaaggcattttcgatttgtgttggtacactgtcgtagagcactacttgatagcccaccgaggcgaagagc
atggaccatgaacgtccgataagaccgctaaatgttaaatattagtataaattttgtatttttttattgacgtttca
cttgatattttttcgttttctttgaaagtcacctgcccacaataccaactttgcattgactggcc
