# Supplementary material for: Conditional embryonic lethality to improve the sterile insect technique in Ceratitis capitata (Diptera: Tephritidae)
Source: BMC Biol. 2009 Jan 27;7:4. doi: 10.1186/1741-7007-7-4 (PMC2662800; doi:10.1186/1741-7007-7-4)
Supplement: Additional file 3 — Raw data of the efficiency test [file 1741-7007-7-4-S3.pdf]

**Additional File 3. Raw data of the efficiency test (Figure 6B).** Raw data on collected eggs, hatched L1 larvae, L3 larvae, pupae, and eclosed adults from WT females crossed to males from homozygous LLs are shown. Four repetitions were carried out. Due to difficulties in the larval count, the number of surviving larvae might be an under-representation.

| | | LL #29 ♀ x WT♂ | LL #66 ♀ x WT♂ | LL #72 ♀ x WT♂ | LL #67 ♀ x WT♂ | LL #68 ♀ x WT♂ | WT ♀ x WT♂ |
|---|---|---|---|---|---|---|---|
| 1. Repetition | eggs | 490 | 576 | 560 | 380 | 540 | 677 |
| | L1 larvae | 107 | 3 | 128 | 0 | 206 | 302 |
| | L3 larvae | 20 | 0 | 8 | 0 | 305 | 450 |
| | pupae | 19 | 0 | 2 | 0 | 208 | 442 |
| | adults | 11 | 0 | 0 | 0 | 0 | 429 |
| | | | | | | | |
| 2. Repetition | eggs | 650 | 565 | 720 | 420 | 569 | 823 |
| | L1 larvae | 116 | 2 | 140 | 0 | 205 | 377 |
| | L3 larvae | 10 | 0 | 10 | 0 | 223 | 489 |
| | pupae | 9 | 0 | 6 | 0 | 202 | 467 |
| | adults | 5 | 0 | 3 | 0 | 0 | 448 |
| | | | | | | | |
| 3. Repetition | eggs | 170 | 700 | 1850 | 690 | 350 | 560 |
| | L1 larvae | 35 | 11 | 365 | 0 | 61 | 236 |
| | L3 larvae | 11 | 0 | 12 | 0 | 98 | 367 |
| | pupae | 11 | 0 | 3 | 0 | 145 | 350 |
| | adults | 8 | 0 | 2 | 0 | 0 | 333 |
| | | | | | | | |
| 4. Repetition | eggs | 189 | 437 | 1200 | 568 | 455 | 351 |
| | L1 larvae | 26 | 2 | 212 | 0 | 145 | 156 |
| | L3 larvae | 22 | 0 | 16 | 0 | 199 | 238 |
| | pupae | 15 | 0 | 2 | 0 | 195 | 223 |
| | adults | 13 | 0 | 1 | 0 | 0 | 209 |
