# Supplementary material for: Conditional embryonic lethality to improve the sterile insect technique in Ceratitis capitata (Diptera: Tephritidae)
Source: BMC Biol. 2009 Jan 27;7:4. doi: 10.1186/1741-7007-7-4 (PMC2662800; doi:10.1186/1741-7007-7-4)
Supplement: Additional file 4 — Primer sequences shown in 5' to 3' orientation [file 1741-7007-7-4-S4.pdf]

Additional File 4. Primer sequences shown in 5´ to 3´ orientation.

mfs-41	GCGGATAACAATTTCACACAGGAAACAGCTATGAC
mfs-42	CCCAGTCACGACGTTGTAAAACGACGGCCAG
mfs-77	GGTTTTCGGATTCTTTGCAATTCACGATG
mfs-78	CGCTCTCGGAAAGATCAGTACG
mfs-79	CCACAGCCACCCGACGCCATTGG
mfs-80	CTTCGAGTGCTCCGTTGAAGATGATAGC
mfs-83	CGGAAGGCGCCAAATTGTCG
mfs-85	GCGAGAGGTTGTTGTATGTCCGGCAC
mfs-104	CATACGTTGAACAAGAGGCAGCCCGC
mfs-108	GTGTAATTGCTGGTCGGTCGACAC
mfs-110	CATAGAAGACACCGGGACCGATCCAG
mfs-111	GCATGCGGAGATCTAAGCTTGGTCGAG
mfs-112	GTCCGTAGAGATCTGCCGCAGCATTGTCC
mfs-113	AGCATTCTAGACATACTGGCCGGCGGAGC
mfs-117	GATCGGCCGGCCTTGGCGCGCCTA
mfs-118	GATCTAGGCGCGCCAAGGCCGGCC
mfs-131	GTCCGTGCGAATTCTGTATGCATAAGTCG
mfs-133	CGTAGGAGCTCTAGACATTTCTGTGATG
mfs-141	TCCTTATTGACCGTACGACCTTGTGGC
mfs-142	GTCCGTGCGAATTCTAAATTCTGAAAGCTATCTGG
mfs-143	TCAGGTCTCTCTAGACATTTTTTTTTTTAATTTTCACAATTCT
mfs-159	CACAGGAGCTGAAGTGCCAACTGATG
mfs-160	CTTCTGTGGTTACTTTTATGAGTTCGCCG
mfs-161	TCCGTCCGCCGTCATATTGG
mfs-162	TAGCCAGATTCCGTTTCACATTC
mfs-170	CTTCGAGTTCGATCACTGCACAATTC
mfs-171	AGTTTTCAGCCGCAGGCTTTCG
mfs-172	CGCATAAACTGGATGTGGCACGCC
mfs-173	ACATTGAAATGTAGTGAAATGGTGGCG
mfs-188	AGCATTCTAGACATATTGGATTTTCAATAAACAAGTATTTC
mfs-189	ACAGTCCATGGCTATCCGTGCTTTCGCTACATTTATC
mfs-190	ACAGTCCATGGCAGTGGTCACTCATCATCCTTCAAAATG
mfs-191	TCAGGTCTCTCTAGACATTTTGATAATTGAACACTTTACCACGCTC
mfs-201	AATTCGTGCCCCAACTGGGGTAACCTTTGAGTTCTCTCAGTTGGGGGCGTAGGGTC
mfs-202	AATTGACCCTACGCCCCCAACTGAGAGAACTCAAAGGTTACCCCAGTTGGGGCACG
mfs-203	CATGGGTGCCCCAACTGGGGTAACCTTTGAGTTCTCTCAGTTGGGGGCGTAGGGTCG
mfs-204	CATGCGACCCTACGCCCCCAACTGAGAGAACTCAAAGGTTACCCCAGTTGGGGCACC
mfs-211	CTAGGGTGCCCCAACTGGGGTAACCTTTGAGTTCTCTCAGTTGGGGGCGTAGGGTCG
mfs-212	CTAGCGACCCTACGCCCCCAACTGAGAGAACTCAAAGGTTACCCCAGTTGGGGCACC
mfs-333	GCTCCTCCAAGAACGTCATC
mfs-334	TGGTGTAGTCCTCGTTGTGG
mfs-335	GTAATACGACTCACTATAGGGCGGCGGTCACGAACTCCAG
mfs-336	GTGAGCAAGGGCGAGGAG
DsRed_F	CCACCACCTGTTCCTGTAGC
DsRed_R	TTGCCTTTCGCCTTATTTTAG
