# Supplementary material for: Conditional embryonic lethality to improve the sterile insect technique in Ceratitis capitata (Diptera: Tephritidae)
Source: BMC Biol. 2009 Jan 27;7:4. doi: 10.1186/1741-7007-7-4 (PMC2662800; doi:10.1186/1741-7007-7-4)
Supplement: Additional file 5 — Statistical analysis [file 1741-7007-7-4-S5.pdf]

Additional File 5. Statistical analysis. ns = non-significant; - = the original data was 0 for all repetitions, statistics are therefore not possible. T-test and chi-test were performed as described in Sokal and Rohlf [1].

(A) Chi-test for the reversibility tests

	stat	df	probability	significance
**Day 1 (+Tc)**	0,0008	1	0,9768	ns
**Day 2 (+Tc)**	0,0067	1	0,9348	ns
**Day 3 (-Tc)**	0,0152	1	0,9020	ns
**Day 4 (-Tc)**	0,0098	1	0,9213	ns
**Day 5 (-Tc)**	0,0120	1	0,9127	ns
**Day 6 (-Tc)**	0,0139	1	0,9060	ns
**Day 7 (-Tc)**	-	-	-	ns
**Day 8 (+Tc)**	0,0314	1	0,8593	ns
**Day 9 (+Tc)**	0,0088	1	0,9251	ns
**Day 10 (+Tc)**	0,0059	1	0,9389	ns

(B) T-test for the efficiency tests.

		stat	df	probability	significance
**LL #29**	L1 larvae	4,9448	2	0,1270	ns
	pupae	0,1624	2	0,8975	ns
	adults	0,1177	2	0,9254	ns
**LL #72**	L1 larvae	2,2391	2	0,2673	ns
	pupae	0,8260	2	0,5604	ns
	adults	1,4728	2	0,3797	ns
**LL #66**	L1 larvae	1,6379	2	0,3489	ns
	pupae	-	-	-	-
	adults	-	-	-	-
**LL #67**	L1 larvae	-	-	-	-
	pupae	-	-	-	-
	adults	-	-	-	-
**LL #68**	L1 larvae	0,6821	2	0,6188	ns
	pupae	0,2245	2	0,8593	ns
	adults	-	-	-	-
**WT**	L1 larvae	1,6911	2	0,3399	ns
	pupae	1,3158	2	0,4137	ns
	adults	1,9443	2	0,3024	ns

(C) T-test for the competition tests. Ratios describe WT females: WT males: transgenic males.

	stat	df	probability	significance
**1:1:0**	0,9017	10	0,4182	ns
**1:1:1 (LL #66)**	0,7000	10	0,5151	ns
**1:1:3 (LL #66)**	1,0101	10	0,3588	ns
**1:1:5 (LL #66)**	1,4861	10	0,1974	ns
**1:1:9 (LL #66)**	1,0613	10	0,3371	ns
**1:1:1 (LL #67)**	0,1265	10	0,9043	ns
**1:1:3 (LL #67)**	0,0690	10	0,9477	ns
**1:1:5 (LL #67)**	1,7320	10	0,1438	ns
**1:1:9 (LL #67)**	1,4029	10	0,2196	ns
**1:10:0**	0,0987	10	0,9261	ns

Reference

1. Sokal RR, Rohlf FJ: Biometry: the principles and practice of statistics in biological research, 3rd edn. New York, NY: W.H. Freeman and Co.; 1995.
